# CO_2_-Induced ATP-Dependent Release of Acetylcholine on the Ventral Surface of the Medulla Oblongata

**DOI:** 10.1371/journal.pone.0167861

**Published:** 2016-12-09

**Authors:** Robert T. R. Huckstepp, Enrique Llaudet, Alexander V. Gourine

**Affiliations:** 1 Centre for Cardiovascular and Metabolic Neuroscience, Neuroscience, Physiology and Pharmacology, University College London, London, United Kingdom; 2 School of Life Sciences, University of Warwick, Coventry, United Kingdom; Ospedale del Cuore G Pasquinucci Fondazione Toscana Gabriele Monasterio di Massa, ITALY

## Abstract

Complex mechanisms that detect changes in brainstem parenchymal *P*CO_2_/[H^+^] and trigger adaptive changes in lung ventilation are responsible for central respiratory CO_2_ chemosensitivity. Previous studies of chemosensory signalling pathways suggest that at the level of the ventral surface of the medulla oblongata (VMS), CO_2_-induced changes in ventilation are (at least in part) mediated by the release and actions of ATP and/or acetylcholine (ACh). Here we performed simultaneous real-time biosensor recordings of CO_2_-induced ATP and ACh release from the VMS *in vivo* and *in vitro*, to test the hypothesis that central respiratory CO_2_ chemosensory transduction involves simultaneous recruitment of purinergic and cholinergic signalling pathways. In anaesthetised and artificially ventilated rats, an increase in inspired CO_2_ triggered ACh release on the VMS with a peak amplitude of ~5 μM. Release of ACh was only detected after the onset of CO_2_-induced activation of the respiratory activity and was markedly reduced (by ~70%) by ATP receptor blockade. In horizontal slices of the VMS, CO_2_-induced release of ATP was reliably detected, whereas CO_2_ or bath application of ATP (100 μM) failed to trigger release of ACh. These results suggest that during hypercapnia locally produced ATP induces or potentiates the release of ACh (likely from the medullary projections of distal groups of cholinergic neurones), which may also contribute to the development and/or maintenance of the ventilatory response to CO_2_.

## Introduction

Breathing is a vital physiological function that maintains constant levels of the arterial and brain *P*CO_2_/pH to support metabolic demands under ever-changing physiological and environmental conditions. Arterial blood gases and pH are monitored by the peripheral chemoreceptors located in the carotid and (in some species) aortic bodies, [1, 2] and central chemoreceptors located within the medulla oblongata [3, 4]. Ventilatory responses to the increases in arterial *P*CO_2_ are largely preserved after peripheral chemodenervation [5] and resection of the pons and dorsal medulla [6], suggesting that the ventral regions of the medulla oblongata are sensitive to, and mediate, the actions of CO_2_ on breathing [7–10]. Current models of central respiratory CO_2_ chemosensitivity have centred around the function of the so-called retrotrapezoid nucleus (RTN) located near the ventral surface of the medulla (VMS) oblongata, as loss of RTN neurons abolishes CO_2_-induced recruitment of the expiratory activity and significantly reduces CO_2_-induced enhancement of the inspiratory activity [11–13].

Early investigations of the signalling mechanisms underlying central respiratory CO_2_ chemosensitivity suggested that at the VMS, acidification-induced changes in breathing are mediated by the release and actions of acetylcholine (ACh) [14, 15]. It was reported recently that ACh is indeed able to increase the excitability of chemosensitive RTN neurones via activation of muscarinic receptors and subsequent inhibition of KCNQ channels [16]. There is also evidence indicating that the central respiratory chemosensory transduction is mediated by the actions of ATP released by the VMS astrocytes [9, 17–21]. Therefore, central respiratory CO_2_ chemosensory transduction may involve simultaneous recruitment of purinergic and cholinergic signalling pathways similar to those employed for chemosensory transduction in the carotid body [22–24]. Here we tested this hypothesis by making simultaneous real-time biosensor recordings of CO_2_-evoked release of ATP and ACh from the VMS in anaesthetised and artificially ventilated rats, and reduced preparations of the VMS *in vitro*. We also determined the effect of ATP receptor blockade on ACh release during systemic hypercapnia.

## Methods

### ATP and ACh biosensors

**Fig 1:**
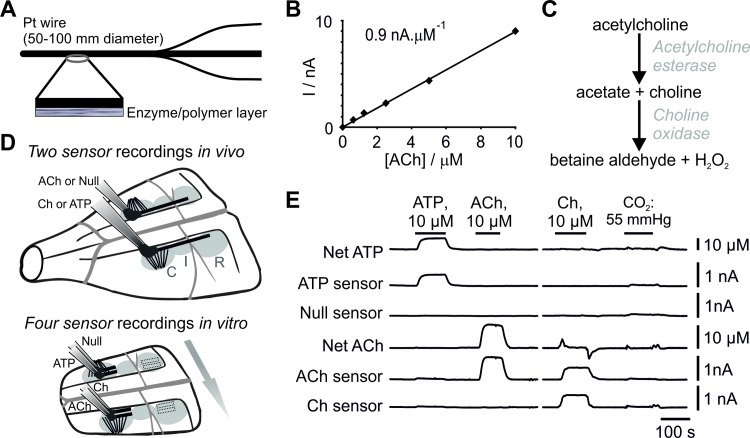
Principles of operation and performance of acetylcholine (ACh) biosensors. (**A**) Schematic of the sensor assembly. (**B**) Calibration curve of a 2 mm ACh biosensor demonstrating linearity of ACh detection in concentrations between 0.5 and 10 μM. (**C**) Enzymatic cascade used to detect ACh. In the presence of ACh, the enzymatic cascade generates H_2_O_2_, which is detected electrochemically. (**D**) Biosensor placements on the ventral medullary surface. *In vivo*, 2 mm sensors were placed in direct contact with the ventral surface of the medulla (VMS) overlaying the rostral (R), intermediate (I) and caudal (C) chemosenitive areas. *In vitro*, 0.5 mm sensors were placed on the VMS within either the rostral or caudal chemosenitive areas. Arrow shows the direction of aCSF flow across the brainstem slice. (**E**) Representative traces illustrating the responses of ATP, null, ACh, and Ch biosensors to ATP and ACh (calibration of 0.5 mm sensors following *in vitro* experiments). Subtracting null sensor current from the ATP biosensor current and subtracting Ch biosensor current from ACh biosensor current produce netATP and netACh signals, respectively.

ATP, ACh, Choline (Ch), and null biosensors were obtained from Sarissa Biomedical Ltd (Coventry, UK). The design and operation of enzymatic ATP biosensors have been described in detail previously [25, 26]. Null sensors, which lack enzymes in the deposition layer, were used to control for the potential release of non-specific electrochemical signals in the experiments involving detection of ATP. ACh sensors utilise two enzymes, acetylcholine esterase and choline oxidase, entrapped within a sol-gel matrix around a Pt wire (50 or 100 μm in diameter and 0.5 or 2 mm long) (Fig 1A and 1C). These enzymes degrade ACh to betaine aldehyde and H_2_O_2_ which is detected electrochemically (Fig 1C). Ch sensors, which only contain choline oxidase in the matrix layer, were used to control for the potential release of choline or non-specific electroactive interferents (Fig 1D). Subtraction of control sensor (null or Ch) currents from ATP and ACh biosensor currents produced the netATP and netACh readings used for the analysis (Fig 1E).

These recording configurations were used for all the experiments except for simultaneous measurements of ATP and ACh release *in vivo* once a sufficient number of controls had been performed to be confident that the sensors, due to their permselective screening layer, do not detect electroactive interferents released during CO_2_ challenges. Furthermore, a sufficient number of tests had also been performed showing that Ch is not detected in the absence of ACh, therefore any detection of Ch by the ACh sensor was secondary to the breakdown of ACh by tissue acetylchloline esterase. Before and after each of the experiments, the sensors were calibrated using 10 μM ATP, ACh, and Ch (Fig 1B and 1E). Sensors were also regularly tested against 10 μM 5-HT to assess the integrity of the sensor permselective screening layer and any reactivity to potential electroactive interferents. Calibrations of sensors used in the *in vivo* experiments were performed in a flow chamber at room temperature. Enzymatic amperometric biosensors have only a small degree of temperature sensitivity. Whilst the absolute increases in ATP and ACh release might have been slightly over-estimated, as all experimental conditions were compared against controls in the same preparation, the relative changes in ATP and ACh concentrations under various conditions were the same. Sensors used in the *in vitro* experiments were calibrated at 33°C in the recording chamber.

### Experimental animals and ethical approval

All experiments were performed in accordance with the European Commission Directive 2010/63/EU (European Convention for the Protection of Vertebrate Animals used for Experimental and Other Scientific Purposes) and the UK Home Office (Scientific Procedures) Act (1986) with project approval from the Institutional Animal Care and Use Committees of the University College London and the University of Warwick

Chemoreception changes with age, and this must be taken into the account when studying the mechanisms underlying the CO_2_ ventilatory response [10]. In this study only adult rats were used as the contribution of CO_2_-induced ATP release to the hypercapnic ventilatory response is well documented in rats of this age range [17, 18, 27, 28].

### *In vivo* preparation

Sixteen male Sprague-Dawley rats (300–340 g) were anaesthetised with sodium pentobarbitone (60 mg.kg^-1^, i.p.). We previously demonstrated that CO_2_-induced release of ATP is preserved in animals anaesthetised with pentobrabitone or urethane [9, 17]. Adequate depth of anaesthesia was monitored through recordings of the respiratory activity (as measured by phrenic nerve discharge), systemic arterial blood pressure and heart rate.

The femoral vein was cannulated for administration of supplemental anaesthetic (sodium pentobarbitone, 10-15 mg.kg^-1^.hr^-1^). The trachea was cannulated and the animal was mechanically ventilated with a tidal volume of ~2 ml and a respiratory frequency of ~60 strokes.min^-1^ with O_2_-enriched air (50% O_2_/50% N_2_). The rat was then placed in a stereotaxic frame and neuromuscular blockade was applied (gallamine triethiodide, 30 mg.kg^-1^, i.v.; with supplemental doses given as required, 3-6 mg.kg^-1^.h^-1^). The VMS was exposed as described previously [9]. Phrenic nerve activity was recorded as a measure of central respiratory drive. The signal was amplified (x20,000), filtered (500–1500 Hz), rectified and smoothed (τ = 50 ms). *P*O_2_, *P*CO_2_ and pH of the arterial blood were measured regularly (every 1–2 h) using a blood gas analyser (Siemens). End-tidal CO_2_ was monitored on-line using a small animal fast-response CO_2_ analyser (model Capstar-100, CWE Inc.). *P*aO_2_ was kept at >100 mmHg to minimize drive from the peripheral chemoreceptors. Body temperature was maintained with a servo-controlled heating pad at 37.0±0.5°C.

### Measurements of CO_2_-induced ATP and ACh release on the VMS *in vivo*

ATP, ACh, Ch, and null biosensors (length of the sensitive tip 2 mm; 100 μm in diameter) were held on a stereotaxic micromanipulator and connected to MicroC potentiostats (WPI). The sensors were bent and the sensing part was laid flat against the VMS and aligned with the pyramidal tracts ~1.5–2.0 mm lateral from the midline (Fig 1D), to maximise the surface area of the biosensor in direct contact with the chemosensitive areas of the VMS including the RTN. Control (null or Ch) sensors were placed in equivalent positions on the contralateral side of the VMS (Fig 1D) as described in detail previously [9, 17] (see Fig 1 in Ref 9). Once the sensors were placed, a period of ~30 mins was allowed until a steady baseline was achieved. To determine the temporal relationship between ATP and/or ACh release on the VMS with CO_2_-evoked increases in respiratory activity, hypocapnic apnoea was induced by mechanical hyperventilation to keep arterial *P*CO_2_ and end-tidal CO_2_ below the apnoeic threshold. CO_2_ was then added to the respiratory mixture until end tidal CO_2_ was ~8% for a period of 3–5 min.

To determine the causal relationship between ATP and ACh release, P2 receptor antagonist pyridoxal-phosphate-6-azophenyl-2',4'-disulfonate (PPADS, 200 μM) was applied to the VMS for 30 min and CO_2_-evoked ACh release was determined. The VMS was washed and after a recovery period of 30–60 min the response to CO_2_ was retested.

To determine the temporal relationship between CO_2_-induced ACh and ATP release, both transmitters were recorded simultaneously using their respective biosensors in the same preparations (n = 8). These recordings were also used to determine the temporal relationship between the onset of ATP release and adaptive increases in the respiratory activity (n = 8). To determine the effect of PPADDS on ACh release, the dual recording configuration of ACh and null sensors was used (n = 8). To determine the temporal relationship between the onset of ACh release and adaptive increases in breathing, the data from both of these experiments were pooled (n = 16).

### *In vitro* slice preparation

Young adult rats (4–6 weeks old; n = 6) were used for the *in vitro* experiments as preparation of the horizontal brainstem slice is significantly easier than in older adult rats. We previously reported [17] that CO_2_-induced ATP release mechanisms are similar in young and older adult rats. The animals were humanely sacrificed by isoflurane overdose. Their brainstems were rapidly dissected free from the skull and placed in a chilled (~5°C) artificial cerebrospinal fluid (aCSF) containing: 124 mM NaCl, 3 mM KCl, 1 mM CaCl_2_, 26 mM NaHCO_3_, 1.25 mM NaH_2_PO_4_, 1 mM MgSO_4_, 10 mM D-glucose (saturated with 95% O_2_/5% CO_2_; pH 7.5) with an additional 10 mM Mg^2+^. A 400 μm thick horizontal slice of the brainstem containing the VMS was prepared as described in detail previously [17, 27], and placed on an elevated grid in a recording chamber superfused with a standard aCSF at 33°C at a rate of 6 ml.min^-1^. The surface of the brainstem slice extended from the pontine border down to the bifurcation of the basilar artery, thus containing almost the entire rostro-caudal extent of the VMS (Fig 1D). Laterally, the surface of the slice extended from the midline to the areas located beyond the lateral border of the facial nucleus on both sides [17, 27] (Fig 1D).

ATP, ACh, Ch, and null biosensors (0.5 mm in length, 50 μm in diameter) were bent and the sensing part was laid flat on the VMS in either the rostral chemosensitive area overlying the RTN or in the caudal chemosensitive region (as no differences in ATP or ACh release were seen under any experimental conditions the data were pooled). The ATP and null sensors were placed on the same side of the VMS juxtaposed to the pyramidal tracts, with ACh and Ch sensors placed in equivalent positions on the contralateral side. To ensure any enzymatic breakdown products from the ATP and ACh sensors did not reach the control sensors (null and Ch), ATP and ACh sensors were placed downstream of the control sensors with respect to the aCSF flow in the recording chamber (Fig 1D). Once the sensors were placed, a period of ~30 mins was allowed until a steady baseline was achieved.

To study the release of ATP and ACh *in vitro*, the VMS slices were exposed to a 5 min chemosensory stimulus followed by a 45 min recovery period. The chemosensory challenge involved replacing normal aCSF with a solution containing: 100 mM NaCl, 50 mM NaHCO_3_, 3 mM KCl, 2 mM CaCl_2_, 1.25 mM NaH_2_PO_4_, 1 mM MgSO_4_, 10 mM Glucose, saturated with 9% CO_2_ (*P*CO_2_ ~55 mmHg, pH 7.5). This chemosensory stimulus was previously demonstrated to induce robust ATP release from the VMS *in vitro* [17, 29, 30]. Following the recovery period, 1 mM ATP was applied to determine the ability of P2 receptor activation to trigger release of ACh. Glycerol (2 mM) was added to all the solutions to enable operation of ATP biosensors. The pH within the recording chamber was monitored with a miniature pH sensitive electrode (Harvard Apparatus).

### Data analysis

*In vivo* records were acquired using *Spike2* software (Cambridge Electronic Design); *in vitro* records we acquired using custom-written software. All data were analysed with OriginPro (OriginLab Corp.). The paired-sample Wilcoxon signed-rank test was used to compare the time delays between the release of ACh and ATP in response to CO_2_. The one-sample Wilcoxon signed rank test was used to analyse whether the peak release of ACh and ATP were different from 0. A one-way repeated measures ANOVA with Dunn-Sidak post-hoc analysis was performed on ranked data to determine the effect of PPADS on ACh release. To exclude the possibility that the effect of PPADS might be masked by generalized run-down of ACh release over time, only experiments where there was at least a partial recovery of ACh release (8/10) were included in the analysis. Data are reported as medians and interquartile ranges (IQR). Differences with p < 0.05 were considered to be significant.

## Results

The ACh sensors had a detection limit of 100 nM ACh, and responded linearly when ACh was present in concentrations of up to 50 μM (Fig 1). Likewise, the Ch sensors had a detection limit of 100 nM Ch, and had a linear response range in concentrations of up to 50 μM (data not shown). The permselective layer blocked the interaction of electroactive interferents (e.g. 5-HT) with the platinum wire of all sensor types, and only small deflections in sensor current were recorded during the application of the modified aCSF used to mimic the chemosensory challenge *in vitro* (Fig 1E). Detection of ACh and Ch by the biosensors was also unaffected in the presence of the P2 receptor antagonist PPADS. Therefore, netACh and netATP signals recorded provided accurate measures of the release of these transmitters.

**Fig 2:**
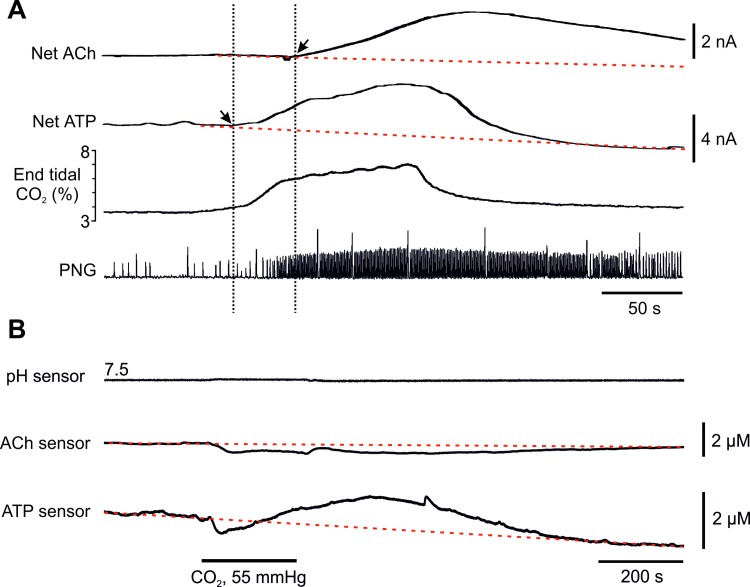
Release of ACh and ATP on the ventral surface of the medulla oblongata during hypercapnia. (**A**) Representative recordings illustrating changes in ACh and ATP concentration on the VMS in response to an increase in the level of inspired CO_2_ in anaesthetised, paralyzed and artificially ventilated rats. Note that ATP release precedes ACh release, which occurs after the onset of CO_2_-induced enhancement of the respiratory activity. Arrows denote when concentrations of ATP and ACh on the VMS start to increase. PNG–phrenic neurogram (arbitrary units). (**B**) Representative recordings illustrating changes in ATP and ACh release from the VMS triggered by CO_2_ *in vitro* (horizontal brainstem slice).

The potential role of ACh in the mechanisms of central respiratory chemosensory transduction was assessed by measuring its release from the VMS in real-time in anaesthetised, paralysed, and artificially ventilated rats (Fig 2). If ACh is one of the key signalling molecules of central chemosensory transduction, its release should be induced by increases in the level of inspired CO_2_, and should precede CO_2_-induced enhancement of the respiratory activity. Increasing inspired CO_2_ was found to be associated with significant ACh release detected on the VMS, which reached a peak amplitude of 4.7 μM (IQR = 4.3 μM; n = 16; P = 0.0004; Fig 2A). Release of ACh was detected 6.6 s (IQR = 11.7 s) after hypercapnia-induced activation of respiration was recorded (n = 8, Fig 2A). Null sensors did not report any signal changes during the chemosensory challenges, and Ch release was not detected in the absence of ACh release. Therefore, the signal recorded by the ACh sensor was due to neither the release of non-specific electroactive interferents nor the detection of Ch.

The time course of ACh release contrasted with that of ATP release, which always preceded CO_2_-induced increases in phrenic nerve activity (Gourine et al., 2005b). Therefore, we next assessed the temporal relationship of the release of these two transmitters, by making simultaneous paired measurements of ATP and ACh release on the VMS (Fig 2A). The onset of ATP release was found to precede CO_2_-induced changes in central respiratory drive by 15.5 s (IQR = 9.1 s), and therefore also the release of ACh (n = 8; P = 0.015; Fig 2A). No ACh release was observed in response to a chemosensory challenge (isohydric hypercapnia) in horizontal slices of the VMS (-0.7 μM; IQR = 1.2 μM; n = 8; P = 0.4), while the CO_2_-induced release of ATP was reliably recorded (1.1 μM; IQR = 1.6 μM; n = 6; P = 0.04; Fig 2B).

**Fig 3:**
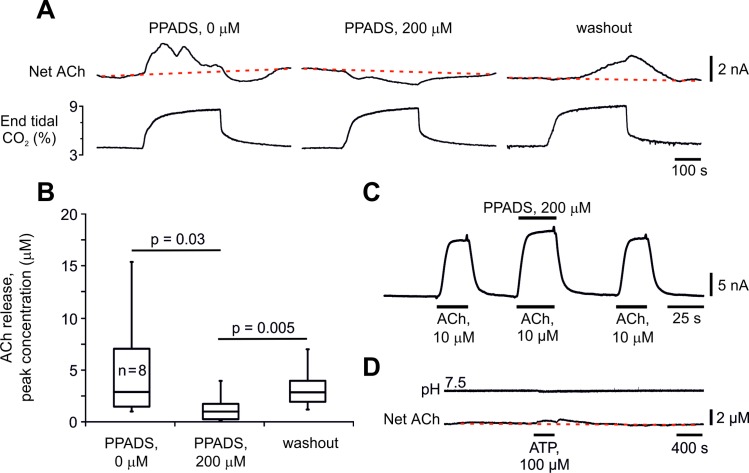
ACh release on the ventral surface of the medulla oblongata during hypercapnia is secondary to the release of ATP. (**A**) Representative recordings obtained sequentially in the same experiment illustrating the effect of P2 receptor antagonist pyridoxal-5’-phosphate-6-azophenyl-2’,4’-disulphonic acid (PPADS) on changes in ACh concentration on the VMS in response to the increases in inspired CO_2_. (**B**) Summary data illustrating CO_2_-induced peak increases in ACh concentration on the VMS in the absence and presence of PPADS and after washout of the drug (n = 8). (**C**) Calibration of ACh biosensors *in vitro* demonstrating that ACh (10 μM)-evoked currents are not reduced in the presence of PPADS (200 μM). (**D**) Representative recordings illustrating lack of changes in ACh release from the VMS in response to bath application of ATP *in vitro* (horizontal brainstem slice).

The temporal differences in the release of these two transmitters *in vivo* suggested that ACh may be released as a downstream consequence of ATP actions. To test this hypothesis, we examined the effect of P2 receptor blockade (through topical application of PPADS) on ACh release at the VMS. PPADS (200 μM) reduced CO_2_-induced release of ACh detected at the VMS by ~70% (from 2.8 μM [IQR 5.6 μM] to 0.9 μM [IQR = 1.4 μM]; n = 8; p = 0.03, Fig 3). Full recovery of ACh release was observed after PPADS washout (2.8 μM [IQR 2.1 μM]; n = 8; P = 0.005 compared to PPADS; P = 0.8 compared to control values; Fig 3). Currents generated by ACh biosensors during *in vitro* calibration with ACh (10 μM) were not reduced in the presence of PPADS (200 μM), indicating that this compound has no significant effect on the biosensor detection system (Fig 3C). In horizontal VMS slices, application of ATP (100 μM) failed to trigger significant ACh release (0.3 μM; IQR = 1.4 μM; n = 8; P = 1.0; Fig 3D), suggesting that either ATP does not directly elicit ACh release and/or the VMS slice does not contain the major source of ACh release observed *in vivo*.

## Discussion

In a series of pioneering studies, Loeschcke and colleagues proposed a key role for ACh in central respiratory chemosensory transduction [7]. This hypothesis was based on the observations that ACh receptor antagonists reduce both the sensitivity of the respiratory network to CO_2_ *in vivo* [14] and the pH-sensitivity of medullary (presumably RTN) neurones *in vitro* [15]. In addition, Dev and Loeschcke also reported that topical applications of ACh or nicotine to pH-sensitive areas of the VMS increase ventilation [14].

Results of the present study show that ACh release occurs too late to be responsible for CO_2_-induced initiation of the respiratory activity from hypocapnic apnoea. Moreover, during systemic hypercapnia, ACh release on the VMS was preceded by ATP release, and was markedly reduced (by ~70%) by ATP receptor blockade following topical application of P2 antagonist. Though ACh release appears to depend on ATP actions, our data suggest that ATP does not itself trigger ACh release, but instead augments it. We saw no evidence of ACh release from the VMS in response to ATP *in vitro*, when ATP was either bath applied (Fig 3), or following ATP release in response to chemosensory challenge (Fig 2B), known to elicit ATP release through connexin 26 hemichannels [17]. There is evidence that cholinergic neurons which reside near the VMS are not involved in either cardiovascular or respiratory control mechanisms [31]. Our observations that CO_2_ triggers ACh release *in vivo*, but not in the reduced *in vitro* preparations of the VMS, suggest that the major source of this ACh release resides outside of the VMS. Therefore, whereas in response to hypercapnia ATP is released by the VMS astrocytes [17, 18, 32], ACh is likely to be derived from the medullary projections of distal groups of cholinergic neurones, e.g. pontomesencephalic tegmental cholinergic complex [33, 34]. This would also account for the observation that although ACh is able to excite RTN neurones, muscarinic blockade has no effect on CO_2_/H^+^-sensitivity of RTN neurones *in vitro* [16].

We also noticed that whilst ATP release closely follows changes in end-tidal CO_2_ *in vivo*, ACh release remains elevated for a more prolonged period. These different kinetics suggest that ATP is released in response to increasing CO_2_ and is responsible for initiation of the respiratory response to hypercapnia, whilst the release of ACh (which occurs later), may act to maintain elevated respiratory activity after the levels of CO_2_ and ATP have returned to baseline.

Current concepts of central respiratory CO_2_ chemosensitivity pose that RTN neurons are intrinsically pH-sensitive (this chemosensitivity is believed to be mediated by proton sensitive GPCRs and K^+^ channels) [35], and play the key role in relaying changes in brainstem parenchymal pH into a modified pattern of breathing [36]. This model, however, cannot explain why RTN neurones are not able to mount an appropriate respiratory response when the pH-sensitivity of neighbouring astrocytes is compromised [37–39]. An alternative (or complimentary) hypothesis proposes that distinct mechanisms underlie the sensitivity of medullary astrocytes to changes in *P*CO_2_ (direct CO_2_-sensing by connexin26 hemichannels) [29] and pH [32, 40] leading to the release of a common signalling molecule (ATP), which mediates CO_2_/H^+^ actions on breathing within the medullary respiratory network, including the RTN [9, 17–20, 27, 28, 41, 42].

## Summary

The results of the present study suggest that during systemic hypercapnia, locally released ATP potentiates the release of ACh (from as of yet unknown sources), and these two transmitters contribute to the initiation and maintenance of the ventilatory response to CO_2_.
